# Vitamin D-Binding Protein, Bioavailable, and Free 25(OH)D, and Mortality: A Systematic Review and Meta-Analysis

**DOI:** 10.3390/nu14193894

**Published:** 2022-09-20

**Authors:** Anna Zhu, Sabine Kuznia, Daniel Boakye, Ben Schöttker, Hermann Brenner

**Affiliations:** 1 Division of Clinical Epidemiology and Aging Research, German Cancer Research Center (DKFZ), Im Neuenheimer Feld 280, 69120 Heidelberg, Germany; 2 Medical Faculty, University of Heidelberg, 69117 Heidelberg, Germany; 3 Network Aging Research, Heidelberg University, 69115 Heidelberg, Germany; 4 Division of Preventive Oncology, German Cancer Research Center (DKFZ) and National Center for Tumor Diseases (NCT), 69120 Heidelberg, Germany; 5 German Cancer Consortium (DKTK), German Cancer Research Center (DKFZ), 69120 Heidelberg, Germany

**Keywords:** vitamin D-binding protein (VDBP), bioavailable 25(OH)D, free 25(OH)D, mortality, systematic review, meta-analysis

## Abstract

Introduction: Observational studies reported inverse associations between serum total 25-hydroxyvitamin D (25(OH)D) concentrations and mortality. Evolving evidence indicated, however, that bioavailable or free 25(OH)D may be even better predictors of mortality. We conducted a systematic review and meta-analysis to summarize the epidemiological evidence on associations of vitamin D-binding protein (VDBP), albumin-bound, bioavailable, and free 25(OH)D, with mortality. Methods: We systematically searched PubMed and Web of Science, up to 27 May 2022. Predictors of interest included serum or plasma concentrations of VDBP, albumin-bound, bioavailable, and free 25(OH)D. Assessed health outcomes were all-cause and cause-specific mortality. We included studies reporting associations between these biomarkers and mortality outcomes. We applied random-effects models for meta-analyses to summarize results from studies assessing the same vitamin D biomarkers and mortality outcomes. Results: We identified twelve eligible studies, including ten on VDBP, eight on bioavailable 25(OH)D, and eight on free 25(OH)D. No study reported on albumin-bound 25(OH)D and mortality. In meta-analyses, the highest levels of bioavailable and free 25(OH)D were associated with 37% (hazard ratio (HR): 0.63, 95% confidence interval (CI): 0.46, 0.87), and 29% (HR: 0.71, 95% CI: 0.53, 0.97) decrease in all-cause mortality, respectively, compared with the lowest levels. These estimates were similar to those for total 25(OH)D (HR: 0.67, 95% CI: 0.56, 0.80) observed in the same studies. Higher VDBP levels were associated with lower all-cause mortality in cancer patient cohorts. However, no such association was observed in general population cohorts. Conclusions: Similar inverse associations of total, bioavailable, and free 25(OH)D with mortality suggest that bioavailable and free 25(OH)D do not provide incremental value in predicting mortality.

## 1. Introduction

Many studies and several meta-analyses have quite consistently shown that lower serum levels of total 25-hydroxyvitamin D (25(OH)D) concentrations are associated with higher mortality [1,2]. However, the association is not linear, and increased mortality seems to be essentially confined to those with vitamin D insufficiency and particularly those with vitamin D deficiency. These patterns have been corroborated in a recent very large meta-analysis comprising more than 500,000 adults from 33 prospective cohort studies, which provided additional support for a causal relationship between total 25(OH)D concentrations and mortality among participants with low vitamin D status by Mendelian Randomization analyses [3].

Approximately 85–90% of total 25(OH)D is bound to vitamin D-binding protein (VDBP) [4]. The remaining 25(OH)D, known as bioavailable 25(OH)D, includes loosely albumin-bound 25(OH)D and free 25(OH)D, which constitute 10–15%, and less than 1% of total 25(OH)D, respectively [5,6]. Some authors suggested that bioavailable or free 25(OH)D may be better biomarkers of vitamin D status and predictors of its health consequences than total 25(OH)D [6,7]. For instance, although Black Americans had much lower levels of total 25(OH)D than White Americans in a cohort of 2085 adults from the United States (US), Black Americans had similar levels of bioavailable 25(OH)D and higher levels of bone mineral density [8].

In order to provide further insight in the prognostic value of the various vitamin D biomarkers for mortality outcomes, we conducted a systematic review and meta-analysis of epidemiological studies on associations of VDBP, albumin-bound, bioavailable, and free 25(OH)D with all-cause and cause-specific mortality, and compared their prognostic values with those of total 25(OH)D.

## 2. Materials and Methods

The protocol of this review was registered at the Prospective Register of Systematic Reviews (PROSPERO, ID: CRD42020172010). The reporting of this review follows the Preferred Reporting Items for Systematic Reviews and Meta-analysis (PRISMA) statement [9].

### 2.1. Literature Search

We conducted a systematic literature search in PubMed and Web of Science to identify eligible studies published up to 27 May 2022. The search strategy is presented in detail in Supplementary Table S1. We also reviewed the reference lists of relevant articles to complement the search for potentially eligible publications. We restricted the review to publications in English.

### 2.2. Study Eligibility

Vitamin D biomarkers included VDBP, albumin-bound, bioavailable, and free 25(OH)D. Health outcomes were all-cause and cause-specific mortality. We included studies, which examined associations between serum or plasma concentrations of at least one of the vitamin D biomarkers with mortality, and reported relevant risk estimates, like relative risks (RRs), hazard ratios (HRs), or odds ratios (ORs). Our review was not restricted by study designs. Both observational studies and randomized clinical trials could be included if they were eligible.

We excluded studies if they were only available as abstracts or posters but not full texts; did not report estimates of associations; were focusing on participants with critical illness, younger than 18 years old, or pregnant women whose production and metabolism of vitamin D would be expected to be different from the general adult population.

### 2.3. Data Extraction and Quality Assessment

Two investigators (AZ and SK) independently extracted data from the eligible studies, using pre-designed data extraction forms. We extracted descriptive characteristics of eligible studies, including authors, publication year, country, type of study population (e.g., general population or patients with specific diseases), sample size, sex, age, vitamin D biomarkers, covariates adjusted for, follow-up time, and mortality endpoints. In addition, we extracted concentrations of vitamin D biomarkers, and estimates of associations of vitamin D biomarkers with mortality, including HRs and 95% confidence intervals (CIs). Although total 25(OH)D was not among the vitamin D biomarkers of primary interest, data on associations of 25(OH)D with the mortality outcomes (where reported) were extracted for comparison and reported along with data on associations of the more specific vitamin D biomarkers with the mortality outcomes from the same studies. Among the eligible studies, some used the unit of ng/mL to indicate total 25(OH)D concentrations while others used nmol/L. In order to make the comparison among different studies easier for the readers, we presented all results in a uniform manner after pertinent transformation (1 ng/mL total 25(OH)D = 2.5 nmol/L total 25(OH)D). Studies reporting only on total 25(OH)D but not on the biomarkers of specific interest were not included in our systematic review.

Two investigators (AZ and SK) independently conducted the quality assessment. The Newcastle-Ottawa Scale was applied to evaluate the quality and risk of bias of eligible studies [10]. As no eligible randomized clinical trial was identified, all of them were observational studies. More details of the quality assessment criteria and corresponding scores are shown in Supplementary Table S2. Assessment scores in the Newcastle-Ottawa Scale have a theoretical range from zero to nine. Higher scores indicate higher quality and lower risk of bias.

### 2.4. Data Synthesis

Twelve eligible studies reported HRs and 95% Cls on associations of any vitamin D biomarker of interest with mortality. Since the eligible studies used different cut-off points to categorize concentrations of vitamin D biomarkers, we focused on comparisons of the highest with the lowest exposure categories. For meta-analyses, the extracted HRs were log-transformed and their standard errors were calculated. We assessed heterogeneity by the *I*^2^ statistic. Due to the small number of eligible studies and high heterogeneity among the included studies, we used random-effects models for meta-analyses of extracted HRs. Due to the small number of eligible studies which reported on mortality from specific causes, we conducted meta-analyses only for all-cause and cancer mortality. Additionally, meta-analyses were stratified by participant characteristics, such as general population, cancer patients, and other patients. Funnel plots were drafted to evaluate potential publication bias (see Supplementary Figures S1 and S2). We performed the meta-analyses using the meta package in R software (version 3.5.3. R Foundation for Statistical Computing, Vienna, Austria). All *p* values are two-sided, and the level of significance was set at 0.05.

## 3. Results

### 3.1. Literature Search

**Figure 1:**
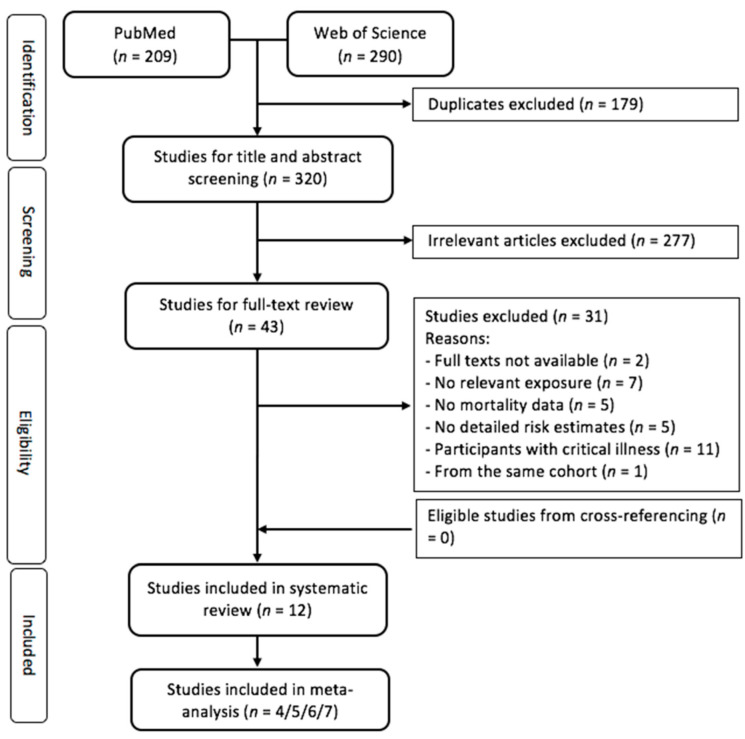
Flow diagram for screening and selecting the eligible studies.

Figure 1 presents the flow chart of the literature search. There were 320 records in the initial search after excluding duplicates. After title and abstract screening, 43 articles were eligible for full-text review. Twelve eligible studies were identified in the systematic review. The number of eligible studies included in the meta-analyses varied, ranging from four to seven, depending on specific vitamin D biomarkers and cause of mortality.

### 3.2. Study Characteristics

Table 1 reports characteristics of the twelve eligible studies. They were all cohort studies that were published from 2013 to 2022. Only two studies recruited participants from the general population [11,12]. Seven studies focused on cancer patients [13,14,15,16,17,18,19] and three on patients with other diseases, i.e., coronary artery disease, chronic obstructive pulmonary disease, and coronavirus disease 2019 (COVID-19) [20,21,22]. Geographically, six studies were from Europe [11,12,16,17,21,22], five from China [13,15,18,19,20], and one from the United States [14]. The sample size ranged from 148 to 5899. The by far largest study was a general population cohort study from Germany [12]. As for sex distribution, two studies included men only [11,17], and the others examined both sexes. Six out of twelve studies [11,12,14,17,20,21] had five years or longer follow-up (up to 20 years). Ten [12,13,14,16,17,18,19,20,21,22], eight [12,13,14,15,18,19,20,22], and eight [11,12,13,14,15,18,20,22] studies examined associations of VDBP, bioavailable, and free 25(OH)D with mortality, respectively. Covariates adjusted for varied to some extent between studies, but most studies adjusted for age, sex, body mass index, and smoking, and half of the studies also adjusted for the time (season) of the blood draw. The summary of risk of bias is shown in Supplementary Table S2. Quality scores ranged from 6 to 9, with a median at 8. The most frequent quality concern referred to adequacy of follow-up.

### 3.3. Vitamin D Biomarkers and Mortality

Two studies reported associations between vitamin D biomarkers and mortality among the general population [11,12] (Table 2). Both studies reported null associations between VDBP and mortality, and inverse associations of total and free 25(OH)D levels with mortality [11,12]. Zhu et al. further reported that bioavailable 25(OH)D levels were inversely associated with mortality [12]. Associations of free and bioavailable 25(OH)D with mortality were very similar to those of total 25(OH)D.

Seven studies reported associations between vitamin D biomarkers and mortality among cancer patients, including three studies among lung cancer patients, two among colorectal cancer patients, and one each among patients with liver cancer and diffuse large B-cell lymphoma [13,14,15,16,17,18,19] (Table 3). Although one of the colorectal cancer patient cohorts [14] and one of the lung cancer patient cohorts (the by far smallest one with 26 deaths overall [16]) showed inverse associations between VDBP levels and mortality, no such association was seen in the other studies [13,17,18,19]. Peng et al. found rather consistent inverse associations between total, bioavailable, and free 25(OH)D and mortality among lung cancer patients [18], as did Chen et al. between total and bioavailable 25(OH)D and mortality among patients with diffuse large B-cell lymphoma [19]. By contrast, quite heterogeneous, partly inconsistent, and null associations were found between these vitamin D biomarkers and mortality in the other cancer patient cohorts [13,15].

Three studies reported associations between vitamin D biomarkers and mortality among patients with other diseases [20,21,22] (Table 4). No clear patterns were seen in the smaller studies among COPD and COVID-19 patients (*n* = 426 and 472, respectively) [21,22]. In the larger study among patients with coronary artery disease (*n* = 1387), Yu et al. showed inverse associations of bioavailable and free 25(OH)D with both all-cause and coronary artery disease mortality [20]. Inverse associations were also reported for total 25(OH)D. However, these associations seemed somewhat weaker and did not reach statistical significance.

### 3.4. Meta-Analyses

Table 5 presents the results of meta-analyses of associations of the highest versus lowest levels of VDBP, total, bioavailable, and free 25(OH)D with all-cause mortality. There was no association between VDBP levels and all-cause mortality (HR: 0.83, 95% CI: 0.65, 1.07). Compared with the lowest levels, the highest levels of bioavailable and free 25(OH)D were associated with 37% (HR: 0.63, 95% CI: 0.46, 0.87) and 29% (HR: 0.71, 95% CI: 0.53, 0.97) decrease in all-cause mortality, respectively. These estimates of reduced mortality were very similar to the corresponding estimate for total 25(OH)D (HR: 0.67, 95% CI: 0.56, 0.80). In the studies among cancer patient cohorts, higher VDBP levels were associated with lower all-cause mortality (HR 0.65, 95% CI: 0.51, 0.82), but such an association was not seen in the general population cohorts (HR 1.03, 95% CI: 0.81, 1.30) and the meta-analysis across all studies.

Table 6 shows the results of meta-analyses of associations with cancer mortality for the highest versus lowest levels of VDBP, total, bioavailable, and free 25(OH)D. Although all of the summary HRs across all studies were below 1 (ranging from 0.81 to 0.94), none of the associations reached statistical significance. Very similar patterns were seen when the meta-analyses were restricted to the cancer patient cohorts.

Funnel plots of the studies included in the meta-analyses do not point to any major publication bias (Supplementary Figures S1 and S2).

## 4. Discussion

Our systematic review and meta-analysis synthesized available evidence on associations of VDBP, bioavailable, and free 25(OH)D with mortality. In the meta-analysis of six cohorts with a total of 9647 participants, participants with the highest levels of bioavailable and free 25(OH)D had 37% and 29% lower all-cause mortality, respectively, compared to those with the lowest levels. These inverse associations with mortality were very similar to inverse associations of total 25(OH)D with mortality observed in the same cohorts. An inverse association of VDBP with mortality was seen in the cancer patient cohorts.

Bioavailable and free 25(OH)D have received increased attention as biomarkers of vitamin D status in recent years. The free hormone hypothesis suggests that hormones that are not bound to high-affinity carrier proteins may easily diffuse through cell membranes for biological activity [15]. Free 25(OH)D, which freely circulates, and 25(OH)D that is loosely bound to albumin, are known as bioavailable 25(OH)D. These forms of vitamin D may dissociate and perform biological actions more rapidly in dynamically perfused tissues [15]. However, the concentrations of bioavailable and free 25(OH)D are highly correlated with those of total 25(OH)D [23], even though they make up less than 15% and 1% of total 25(OH)D. This suggests that total 25(OH)D, which may be more reliably determined by established laboratory methods and whose associations with a broad range of health outcomes have been established by an extensive volume of research, may be an excellent surrogate marker even for bioavailable and free 25(OH)D status. Thus, measurements of bioavailable and free 25(OH)D concentrations may not provide relevant incremental value with respect to mortality prediction compared to total 25(OH)D. Nevertheless, further research based on larger studies is required to enhance the scarce empirical evidence on specific contributions of bioavailable and free 25(OH)D as markers of health relevant vitamin D deficiency.

A particular challenge in that respect is the reliable measurement of bioavailable and free 25(OH)D levels. Methods to determine concentrations of these biomarkers have been heterogeneous, and include both direct measurements and methods to derive concentrations from total 25(OH)D, VDBP, and albumin levels, and their affinity constants depending on the VDBP genotypes. To what extent the various measurements or derivations are reliable and comparable is uncertain. For example, evidence has shown that calculated free 25(OH)D concentrations were lower than directly measured ones, especially among participants with specific physical conditions [24]. In addition, it is hard to accurately measure free 25(OH)D concentrations because of its low concentrations and physicochemical behavior [5].

The inverse associations of total, bioavailable, and free 25(OH)D with mortality seen in our meta-analyses are consistent with meta-analyses of the much larger volume of studies that assessed associations of total 25(OH)D with mortality [25]. It is important to note that such inverse associations seen in observational studies do not necessarily reflect causal associations, even though the majority of studies carefully adjusted for a range of relevant potential confounders. In particular, the observed inverse associations may also partly reflect inverse causality, as 25(OH)D levels may decrease in the course of severe, life threatening diseases [4]. Nevertheless, results of a very large-scale Mendelian Randomization study suggested a causal role of very low vitamin D levels for increased mortality, whereas no such evidence was found for vitamin D levels in the normal and supra-normal range [3].

An interesting finding in the study by Yuan et al. [14] and in our meta-analysis of three studies conducted among cancer patient cohorts is the inverse association between VDBP levels and all-cause mortality among cancer patients which was not observed in the general population cohorts. VDBP levels are strongly genetically determined [23]. If and to what extent their association with mortality among cancer patients can be confirmed in other cancer cohorts and has clinical relevance should be determined in future research.

Our study has several strengths. To our knowledge, it is the first systematic review and meta-analysis to summarize epidemiological evidence on associations of VDBP, bioavailable, and free 25(OH)D with mortality, and to compare their prognostic values with total 25(OH)D. We developed a comprehensive search strategy for selecting the eligible studies, and rigorously adhered to guidelines for conducting and reporting a systematic review.

However, a number of limitations also require careful consideration. Firstly, due to lack of individual data from eligible studies, our review did not conduct individual participant data meta-analysis. This restricted the possibility of subgroup analyses by key characteristics that influence concentrations of vitamin D biomarkers. Secondly, despite a quite comprehensive literature search strategy, we cannot rule out the possibility of having missed one or several eligible studies, especially if they were reported in languages other than English. Thirdly, although interest in the role of specific vitamin D biomarkers for health outcomes is evolving, the number and size of cohort studies assessing the associations of bioavailable and free 25(OH)D with mortality are still very limited. Fourthly, diverse categorization of vitamin D biomarker concentrations, adjustment for different sets of covariates, and considerable heterogeneity in the characteristics of participants among the eligible studies limit the comparability of results across studies. Although we extracted risk estimates (hazard ratios and 95% CI) from full-adjusted regression models among the eligible studies and applied them in the meta-analysis, factors not adjusted for in the original studies could not be taken into account. Fifthly, cause-specific mortality was reported quite heterogeneously, i.e., for different causes of deaths across studies, which limited the possibility to conduct cause-specific meta-analyses of cancer mortality. Lastly, eleven out of twelve eligible studies were conducted in China and Europe, which limits generalizability to populations from other parts of the world.

## 5. Conclusions

Although associations of VDBP, bioavailable, and free 25(OH)D with health outcomes have become a major field of research in recent years, the number of studies assessing their associations with all-cause and cause-specific mortality is still quite limited. Nevertheless, this systematic review and meta-analysis gathered evidence showing that associations of bioavailable and free 25(OH)D with mortality are quite consistent with and similar to those observed for total 25(OH)D. Another interesting finding was the inverse association of VDBP levels with all-cause mortality among cancer patient cohorts but not in general population cohorts. Further research should address the associations of the various vitamin D biomarkers with mortality and other major health outcomes in larger and more diverse populations and evaluate if and to what extent measurement of specific vitamin D biomarkers may be relevant for clinical management of vitamin D deficiency.

## Figures and Tables

**Table 1:** Characteristics of the included studies.

Participants	First Author, Year	Country	Sample Size (Deaths/Total)	Age (Years)		Sex (% Fem)	Follow-Up (Years)	Predictor				Covariates Adjusted for					
				Range	Mean			VDBP	25(OH)D			Age	Sex	BMI	SMK	TIME	OTH
									Total	BIO	Free						
**General** **population**	Dejaeger 2021 [11]	Europe	469/1915	NA	60.1	0	12.3 ^a^	√	√		√	√		√	√	√	^d^
	Zhu 2022 [12]	Germany	1739/5899	49–75	62.3	56.1	17.1 ^a^	√	√	√	√	√	√	√	√	√	^e^
**Cancer patients**																	
Colorectal	Yang 2017 [13]	China	87/206	30–85	63.0 ^c^	36.4	3.8 ^a^	√	√	√	√	√		√	√		^f^
Colorectal	Yuan 2020 [14]	US	328/603	30–75	71.3	59.2	12.4 ^a^	√	√	√	√	√	√	√			^g^
Liver	Fang 2020 [15]	China	430/1031	NA	53.0	11.8	2.0 ^a^		√	√	√ *	√	√	√		√	^h^
Lung	Turner 2013 [16]	UK	26/148	NA	66.8	41.4	4.1 ^b^	√				√	√		√		^i^
Lung	Anic 2014 [17]	Finland	428/500	50–69	68.4	0	20.0 ^c^	√	√			√	√	√	√	√	^j^
Lung	Peng 2022 [18]	China	179/395	NA	63.0	36.2	2.7 ^a^	√	√	√	√ *	√	√	√	√		^k^
DLBCL	Chen 2020 [19]	China	NA/332	>60 years: 39.8%		46.7	2.9 ^a^	√	√	√							^l^
**Other patients**																	
CAD	Yu 2018 [20]	China	205/1387	40–85	63.2	34.9	6.7 ^a^		√	√	√ *	√	√	√	√	√	^m^
COPD	Persson 2015 [21]	Norway	69/426	40–76	63.5	39.9	5.0 ^b^	√	√			√	√		√	√	^n^
COVID-19	Subramanian 2022 [22]	UK	112/472	19–98	66.9	43.2	NA	√	√	√	√	√	√				^o^

Abbreviations: 25(OH)D: 25-hydroxyvitamin D; BIO: bioavailable; BMI: body mass index; CAD: coronary artery disease; COPD: chronic obstructive pulmonary disease; COVID-19: coronavirus disease 2019; DLBCL: diffuse large B-cell lymphoma; fem: females; NA: not available; OTH: others; SMK: smoking; TIME: time (season) of blood draw; US: United States; UK: United Kingdom; VDBP: vitamin D-binding protein. Footnotes: ^a^ median. ^b^ mean. ^c^ The specific number is not stated. The follow-up was up to 20 years. ^d^ study center, alcohol, physical activity, estimated glomerular filtration rate, number of comorbidities. ^e^ school education, physical activity, regular intake of multivitamin supplements, fish consumption. ^f^ stage, hypertension, diabetes, cell differentiation, albumin. ^g^ stage, physical activity, grade of tumor differentiation, location of primary tumor, year of diagnosis, season of blood collection. ^h^ stage, estimated glomerular filtration rate, C-reactive protein, cancer treatment. ^i^ stage, total 25(OH)D levels. ^j^ stage, family history of lung cancer, total daily intake of energy, calcium, fat, total serum cholesterol, daily alcohol intake. ^k^ drinking status, chronic obstructive pulmonary disease, season of blood-drawing, histology, surgery, carcinoembryonic antigen, neuron-specific enolase, albumin, total cholesterol, neutrophil-to-lymphocyte ratio, lymphocyte-to-monocyte ratio, radiotherapy, targeted therapy. ^l^ Eastern Cooperative Oncology Group Score, lactic dehydrogenase level, tumor necrosis factor-α level, Ann Arbor stage, β2-microglobulin level, albumin. ^m^ hypertension, diabetes, total cholesterol, high-density lipoprotein cholesterol, extent of coronary artery disease, presence or absence of acute coronary artery disease, presence or absence of coronary revascularization, use or nonuse of statins, angiotensin-converting enzyme inhibitors/angiotensin receptor blockers, β-blockers, leisure-time physical activity, estimated glomerular filtration rate, calcium, parathyroid hormone, C-reactive protein. ^n^ vitamin D supplements, body composition, number of exacerbations the last year before baseline. ^o^ chronic kidney disease, neurological disease. * indicated direct measurement of free 25(OH)D concentrations.

**Table 2:** Associations of VDBP, total, bioavailable, and free 25(OH)D with mortality among the general population.

Study	Cause of Mortality	VDBP		Total 25(OH)D		Bioavailable 25(OH)D		Free 25(OH)D	
		(μg/mL)	HR (95% CI)	(ng/mL)	HR (95% CI)	(ng/mL)	HR (95% CI)	(pg/mL)	HR (95% CI)
Dejaeger 2021 [11]	All-cause	293.6 (36.7) ^a^	Qi1: Ref ^b^	16.8 (8.9) ^a^	Qi1: Ref ^b^	NA	NA	4.3 (2.3) ^a^	Qi1: Ref ^b^
			Qi2: 0.99 (0.70, 1.40)		Qi2: 0.77 (0.53, 1.12)				Qi2: 0.94 (0.61, 1.47)
			Qi3: 0.94 (0.67, 1.33)		Qi3: 0.82 (0.56, 1.19)				Qi3: 0.93 (0.60, 1.45)
			Qi4: 0.94 (0.67, 1.33)		Qi4: 0.56 (0.38, 0.81)				Qi4: 0.65 (0.42, 1.01)
			Qi5: 1.23 (0.88, 1.75)		Qi5: 0.49 (0.34, 0.72)				Qi5: 0.48 (0.31, 0.75)
Zhu 2022 [12]	All-cause	Q1: 37.8–283.9 Q2: 283.9–314.3 Q3: 314.4–349.6 Q4: 349.7–600.0	Q1: Ref	Q1: 2.8–13.4 Q2: 13.4–17.8 Q3: 17.8–24.0 Q4: 24.0–50.0	Q1: Ref	Q1: 0.3–1.6 Q2: 1.6–2.2 Q3: 2.2–3.1 Q4: 3.1–7.0	Q1: Ref	Q1: 0.6–3.6 Q2: 3.6–4.9 Q3: 4.9–6.9 Q4: 6.9–14.0	Q1: Ref
			Q2: 0.90 (0.79, 1.02)		Q2: 0.80 (0.70, 0.90)		Q2: 0.87 (0.76, 0.99)		Q2: 0.86 (0.76, 0.98)
			Q3: 0.90 (0.79, 1.03)		Q3: 0.72 (0.63, 0.82)		Q3: 0.74 (0.64, 0.84)		Q3: 0.74 (0.65, 0.85)
			Q4: 0.95 (0.83, 1.09)		Q4: 0.64 (0.55, 0.73)		Q4: 0.67 (0.58, 0.77)		Q4: 0.70 (0.60, 0.80)
	CVD		Q1: Ref		Q1: Ref		Q1: Ref		Q1: Ref
			Q2: 0.84 (0.67, 1.06)		Q2: 0.90 (0.72, 1.12)		Q2: 0.91 (0.73, 1.14)		Q2: 0.99 (0.79, 1.24)
			Q3: 0.94 (0.75, 1.18)		Q3: 0.77 (0.61, 0.97)		Q3: 0.80 (0.63, 1.01)		Q3: 0.84 (0.66, 1.06)
			Q4: 0.92 (0.73, 1.17)		Q4: 0.64 (0.50, 0.83)		Q4: 0.64 (0.49, 0.82)		Q4: 0.71 (0.55, 0.91)
	Cancer		Q1: Ref		Q1: Ref		Q1: Ref		Q1: Ref
			Q2: 0.85 (0.68, 1.07)		Q2: 0.76 (0.61, 0.96)		Q2: 0.76 (0.60, 0.96)		Q2: 0.71 (0.56, 0.90)
			Q3: 0.89 (0.70, 1.11)		Q3: 0.82 (0.65, 1.03)		Q3: 0.72 (0.57, 0.91)		Q3: 0.67 (0.53, 0.85)
			Q4: 0.99 (0.79, 1.24)		Q4: 0.76 (0.60, 0.97)		Q4: 0.80 (0.63, 1.02)		Q4: 0.81 (0.64, 1.02)
	Respiratory disease		Q1: Ref		Q1: Ref		Q1: Ref		Q1: Ref
			Q2: 0.73 (0.41, 1.30)		Q2: 0.60 (0.35, 1.04)		Q2: 0.60 (0.34, 1.04)		Q2: 0.61 (0.35, 1.07)
			Q3: 0.67 (0.37, 1.22)		Q3: 0.53 (0.29, 0.94)		Q3: 0.49 (0.28, 0.87)		Q3: 0.54 (0.31, 0.96)
			Q4: 1.08 (0.62, 1.86)		Q4: 0.39 (0.20, 0.74)		Q4: 0.35 (0.18, 0.67)		Q4: 0.37 (0.19, 0.70)

Abbreviations: Cardiovascular disease (CVD); CI: confidence interval; HR: hazard ratio; NA: not available; Q1: quartile 1; Q2: quartile 2; q3: Quartile 3; Q4: quartile 4; Qi1: quintile 1; Qi2: quintile 2; Qi3: quintile 3; Qi4: quintile 4; Qi5: quintile 5; Ref: reference; VDBP: vitamin D-binding protein. Footnotes: ^a^ mean and standard deviation. ^b^ Transformation of risk estimates to make the direction of associations consistent with other studies.

**Table 3:** Associations of VDBP, total, bioavailable, and free 25(OH)D with mortality among cancer patients.

Study	Cause of Mortality	VDBP		Total 25(OH)D		Bioavailable 25(OH)D		Free 25(OH)D	
		(μg/mL)	HR (95% CI)	(ng/mL)	HR (95% CI)	(ng/mL)	HR (95% CI)	(pg/mL)	HR (95% CI)
**Patients with colorectal cancer**									
Yang 2017 [13]	CRC	L: <159	L: Ref	L: <6.2	L: Ref	L: <0.58	L: Ref	L: <0.01	L: Ref
		M: 159–310	M: 1.46 (0.81, 2.66)	M: 6.2–29.9	M: 1.18 (0.72, 1.94)	M: 0.58–1.03	M: 0.81 (0.33, 1.99)	M: 0.01–0.02	M: 0.24 (0.12, 0.50)
		H: >310	H: 2.01 (0.92, 4.42)	H: >29.9	H: 1.79 (0.90, 3.56)	H: >1.03	H: 0.40 (0.082, 1.93)	H:>0.02	H: 0.44 (0.24, 0.82)
Yuan 2020 [14]	All-cause	Q1: 125.2 Q2: 213.5 Q3: 274.6 Q4: 383.5 ^a^	Q1: Ref	Q1: 15.6 Q2: 23.7 Q3: 29.4 Q4: 40.5 ^a^	Q1: Ref	Q1: 1.8 Q2: 2.9 Q3: 3.9 Q4: 6.5 ^a^	Q1: Ref	Q1: 4.5 Q2: 7.0 Q3: 9.4 Q4: 15.8 ^a^	Q1: Ref
			Q2: 0.77 (0.57, 1.06)		Q2: 1.18 (0.84, 1.65)		Q2: 1.11 (0.78, 1.59)		Q2: 1.18 (0.82, 1.69)
			Q3: 0.69 (0.50, 0.96)		Q3: 1.13 (0.80, 1.59)		Q3: 1.12 (0.78, 1.61)		Q3: 1.11 (0.77, 1.59)
			Q4: 0.58 (0.41, 0.80)		Q4: 0.72 (0.49, 1.05)		Q4: 1.19 (0.82, 1.73)		Q4: 1.36 (0.94, 1.95)
	CRC		Q1: Ref		Q1: Ref		Q1: Ref		Q1: Ref
			Q2: 0.76 (0.50, 1.15)		Q2: 1.22 (0.77, 1.93)		Q2: 1.07 (0.66, 1.71)		Q2: 1.18 (0.73, 1.90)
			Q3: 0.73 (0.48, 1.11)		Q3: 1.45 (0.92, 2.30)		Q3: 1.01 (0.61, 1.65)		Q3: 1.05 (0.64, 1.70)
			Q4: 0.58 (0.37, 0.91)		Q4: 0.57 (0.34, 0.97)		Q4: 1.26 (0.77, 2.06)		Q4: 1.35 (0.83, 2.18)
	5-year overall survival		Q1: Ref		Q1: Ref		Q1: Ref		Q1: Ref
			Q2: 0.74 (0.50, 1.10)		Q2: 0.92 (0.60, 1.40)		Q2: 1.07 (0.68, 1.69)		Q2: 1.03 (0.65, 1.64)
			Q3: 0.68 (0.46, 1.01)		Q3: 1.05 (0.69, 1.59)		Q3: 1.03 (0.65, 1.63)		Q3: 0.97 (0.61, 1.53)
			Q4: 0.50 (0.32, 0.76)		Q4: 0.48 (0.30, 0.78)		Q4: 1.14 (0.71, 1.82)		Q4: 1.29 (0.82, 2.02)
**Patients with liver cancer**									
Fang 2020 [15]	All-cause	NA	NA	Q1: ≤27.3 Q2: 27.3–34.6 Q3: 34.6–43.6 Q4: >43.6	Q1: Ref	Q1: ≤1.73 Q2: 1.73–2.12 Q3: 2.12–2.56 Q4: >2.56 ^b^	Q1: Ref	Q1: ≤4.62 Q2: 4.62–5.58 Q3: 5.58–6.71 Q4: >6.71 ^b^	Q1: Ref
					Q2: 0.88 (0.66, 1.18)		Q2: 0.85 (0.66, 1.11)		Q2: 0.83 (0.63, 1.09)
					Q3: 0.97 (0.73, 1.29)		Q3: 0.77 (0.59, 1.00)		Q3: 0.83 (0.63, 1.09)
					Q4: 0.95 (0.72, 1.26)		Q4: 0.71 (0.53, 0.94)		Q4: 0.89 (0.68, 1.18)
	Liver cancer		NA		Q1: Ref		Q1: Ref		Q1: Ref
					Q2: 0.90 (0.66, 1.22)		Q2: 0.80 (0.61, 1.05)		Q2: 0.83 (0.63, 1.10)
					Q3: 0.99 (0.73, 1.32)		Q3: 0.75 (0.57, 0.98)		Q3: 0.79 (0.59, 1.05)
					Q4: 0.97 (0.72, 1.31)		Q4: 0.69 (0.51, 0.93)		Q4: 0.90 (0.68, 1.20)
**Patients with lung cancer**									
Turner 2013 [16]	Lung cancer	Q1: <199	Q1: Ref ^c^	NA	NA	NA	NA	NA	NA
		Q2: 199–332	Q2: 0.55 (0.046, 5.60)						
		Q3: 332–430	Q3: 0.53 (0.044, 5.33)						
		Q4: ≥430	Q4: 0.096 (0.0080, 0.97)						
Anic 2014 [17]	Lung cancer	Q1: <274	Q1: Ref	Season specific quartiles ^d^	Q1: Ref	NA	NA	NA	NA
		Q2: 274–342	Q2: 0.79 (0.59, 1.06)		Q2: 1.08 (0.81, 1.43)				
		Q3: 342–417	Q3: 1.02 (0.76, 1.35)		Q3: 0.97 (0.72, 1.29)				
		Q4: ≥417	Q4: 0.95 (0.71, 1.26)		Q4: 1.18 (0.89, 1.56)				
Peng 2022 [18]	All-cause	T1: ≤181.5 T2: 181.5–222.7 T3: >222.7	T1: Ref	T1: ≤ 16.4 T2: 16.4–23.9 T3: >23.9	T1: Ref	T1: ≤2.21 T2: 2.22–3.40 T3: >3.41	T1: Ref	T1: ≤6.04 T2: 6.05–9.12 T3: >9.13	T1: Ref
			T2: 0.67 (0.46, 0.99)		T2: 0.83 (0.57, 1.21)		T2: 0.63 (0.43, 0.92)		T2: 0.68 (0.47, 1.00)
			T3: 0.74 (0.51, 1.08)		T3: 0.58 (0.40, 0.87)		T3: 0.45 (0.30, 0.67)		T3: 0.49 (0.33, 0.73)
	PFS		T1: Ref		T1: Ref		T1: Ref		T1: Ref
			T2: 0.73 (0.52, 1.02)		T2: 0.69 (0.49, 0.98)		T2: 0.79 (0.56, 1.10)		T2: 0.74 (0.52, 1.05)
			T3: 0.84 (0.60, 1.17)		T3: 0.61 (0.43, 0.86)		T3: 0.56 (0.40, 0.80)		T3: 0.60 (0.42, 0.85)
**Patients with diffuse large B-cell lymphoma**									
Chen 2020 [19]	All-cause	T1: <371 T2: 371–534 T3: >534	T1: Ref ^e^	T1: 0.3–11.4 T2: 11.5–18.6 T3: 18.7–37.8	T1: Ref	T1: 0.094–0.66 T2: 0.66–1.11 T3: 1.11–3.44	T1: Ref	NA	NA
			T2: 0.79 (0.37, 1.66)		T2: 0.90 (0.40, 2.03)		T2: 0.89 (0.39, 2.02)		
			T3: 0.69 (0.33, 1.46)		T3: 0.40 (0.16, 1.03)		T3: 0.21 (0.07, 0.65)		
	PFS		T1: Ref ^e^		T1: Ref		T1: Ref		NA
			T2: 0.75 (0.44, 1.29)		T2: 0.61 (0.34, 1.11)		T2: 0.72 (0.38, 1.35)		
			T3: 0.51 (0.28, 0.91)		T3: 0.27 (0.13, 0.57)		T3: 0.39 (0.20, 0.79)		

Abbreviations: CI: confidence interval; H: high; HR: hazard ratio; L: low; M: middle; NA: not available; PFS: progression free survival; Q1: quartile 1; Q2: quartile 2; q3: Quartile 3; Q4: quartile 4; Ref: reference; T1: tertile 1; T2: tertile 2; T3: tertile 3; VDBP: vitamin D-binding protein. Footnotes: ^a^ mean. ^b^ bioavailable and free 25(OH)D concentrations by quartiles of serum 25(OH)D concentrations. ^c^ Transformation of risk estimates to make the direction of associations consistent with other studies. ^d^ Seasonal specific quartiles (ng/mL): Winter (November–April): Q1: <7.13, Q2: 7.13–10.14, Q3: 10.14–16.35, Q4: ≥16.35; summer (May–October): Q1: <11.82, Q2: 29.5–18.07, Q3: 18.07–24.36, Q4: ≥24.36. ^e^ results of univariate regression.

**Table 4:** Associations of VDBP, total, bioavailable, and free 25(OH)D with mortality among patients with other diseases.

Study	Cause of Mortality	VDBP		Total 25(OH)D		Bioavailable 25(OH)D		Free 25(OH)D	
		(μg/mL)	HR (95% CI)	(ng/mL)	HR (95% CI)	(ng/mL)	HR (95% CI)	(pg/mL)	HR (95% CI)
**Patients with coronary artery disease**									
Yu 2018 [20]	All-cause	Q1: 285 Q2: 210 Q3: 139 Q4: 77 ^a^	NA	Q1: 16.8 Q2: 20.4 Q3: 21.3 Q4: 23.2 ^a^	Q1: Ref ^b^	Q1: ≤2.11 Q2: 2.12–3.17 Q3: 3.18–4.87 Q4: ≥4.88	Q1: Ref ^b^	Q1: 3.17 Q2: 4.19 Q3: 5.14 Q4: 7.41 ^a^	Q1: Ref ^b^
					Q2: 0.67 (0.43, 1.04)		Q2: 0.76 (0.50, 1.15)		Q2: 0.82 (0.54, 1.26)
					Q3: 0.73 (0.47, 1.13)		Q3: 0.76 (0.50, 1.16)		Q3: 0.75 (0.49, 1.14)
					Q4: 0.74 (0.47, 1.14)		Q4: 0.56 (0.37, 0.85)		Q4: 0.61 (0.40, 0.93)
	CAD		NA		Q1: Ref ^b^		Q1: Ref ^b^		Q1: Ref ^b^
					Q2: 0.56 (0.33, 0.97)		Q2: 0.72 (0.41, 1.26)		Q2: 0.81 (0.48, 1.38)
					Q3: 0.66 (0.38, 1.13)		Q3: 0.67 (0.38, 1.18)		Q3: 0.71 (0.42, 1.22)
					Q4: 0.67 (0.39, 1.15)		Q4: 0.39 (0.22, 0.68)		Q4: 0.51 (0.30, 0.87)
**Patients with chronic obstructive pulmonary disease**									
Persson 2015 [21]	All-cause	L: <200	L: Ref	Per 10 ng/mL decrease	0.95 (0.71, 1.26)	NA	NA	NA	NA
		M: 200–299	M: 1.03 (0.60, 1.75)						
		H: ≥300	H: 0.76 (0.28, 2.02)						
**Patients with coronavirus disease 2019**									
Subramanian 2022 [22]	COVID-19	Per 100 μg/mL increase	1.00 (0.97, 1.04)	Qi1: <10	Qi1: Ref ^b^	Qi1: <0.18	Qi1: Ref ^b^	Qi1: <0.62	Qi1: Ref ^b^
				Qi2: 10–19.6	Qi2: 0.79 (0.39, 1.59)	Qi2: 0.18–0.32	Qi2: 0.40 (0.18, 0.86)	Qi2: 0.62–1.08	Qi2: 0.59 (0.28, 1.24)
				Qi3: 20–29.6	Qi3: 0.42 (0.21, 0.85)	Qi3: 0.32–0.52	Qi3: 0.91 (0.42, 1.98)	Qi3: 1.08–1.65	Qi3: 0.87 (0.41, 1.83)
				Qi4: 30–39.6	Qi4: 0.92 (0.46, 1.86)	Qi4: 0.52–0.81	Qi4: 0.59 (0.27, 1.27)	Qi4: 1.65–2.46	Qi4: 0.78 (0.37, 1.63)
				Qi5: ≥40	Qi5: 1.95 (0.98, 3.95)	Qi5: >0.81	Qi5: 0.78 (0.36, 1.69)	Qi5: >2.46	Qi5: 1.16 (0.55, 2.44)

Abbreviations: CI: confidence interval; HR: hazard ratio; NA: not available; PFS: progression free survival; Q1: Quartile 1; Q2: Quartile 2; Q3: Quartile 3; Q4: Quartile 4; Qi1: quintile 1; Qi2: quintile 2; Qi3: quintile 3; Qi4: quintile 4; Qi5: quintile 5; Ref: reference; VDBP: vitamin D-binding protein. Footnotes: ^a^ median of VDBP, total and free 25(OH)D concentrations by quartiles of bioavailable 25(OH)D concentrations. ^b^ Transformation of risk estimates to make the direction of associations consistent with other studies.

**Table 5:** Results of the meta-analyses of VDBP, total, bioavailable, and free 25(OH)D (the highest vs. lowest levels) with all-cause mortality.

Participants	Study	VDBP	Total 25(OH)D	Bioavailable 25(OH)D	Free 25(OH)D
		HR (95% CI)	HR (95% CI)	HR (95% CI)	HR (95% CI)
**General population**	Dejaeger 2021 [11]	1.23 (0.87, 1.73)	0.49 (0.34, 0.71)	-	0.48 (0.31, 0.75)
	Zhu 2022 [12]	0.95 (0.83, 1.09)	0.64 (0.56, 0.74)	0.67 (0.58, 0.77)	0.70 (0.61, 0.81)
	**Subtotal**	**1.03 (0.81, 1.30)**	**0.59 (0.47, 0.75)**	**-**	**0.62 (0.43, 0.87)**
**Cancer patients**					
Colorectal	Yuan 2020 [14]	0.58 (0.42, 0.81)	0.72 (0.49, 1.05)	1.19 (0.82, 1.73)	1.36 (0.94, 1.96)
Liver	Fang 2020 [15]	-	0.95 (0.72, 1.26)	0.71 (0.53, 0.95)	0.89 (0.68, 1.17)
Lung	Peng 2022 [18]	0.74 (0.51, 1.08)	0.58 (0.39, 0.86)	0.45 (0.30, 0.67)	0.49 (0.33, 0.73)
DLBCL	Chen 2020 [19]	0.69 (0.33, 1.45)	0.40 (0.16, 1.01)	0.21 (0.07, 0.64)	-
	**Subtotal**	**0.65 (0.51, 0.82)**	**0.71 (0.53, 0.96)**	**0.60 (0.33, 1.10)**	**0.84 (0.48, 1.49)**
**Other patients**					
CAD	Yu 2018 [20]	-	0.74 (0.48, 1.15)	0.56 (0.37, 0.85)	0.61 (0.40, 0.93)
COPD	Persson 2015 [21]	0.76 (0.28, 2.04)	-	-	-
	**Subtotal**	-	-	-	-
**All studies**		**0.83 (0.65, 1.07)**	**0.67 (0.56, 0.80)**	**0.63 (0.46, 0.87)**	**0.71 (0.53, 0.97)**

Abbreviations: CAD: coronary artery disease; CI: confidence interval; COPD: chronic obstructive pulmonary disease; DLBCL: diffuse large B-cell lymphoma; HR: hazard ratio; VDBP: vitamin D-binding protein.

**Table 6:** Results of the meta-analyses of VDBP, total, bioavailable, and free 25(OH)D (highest vs. lowest levels) with cancer mortality.

Participants	Study	VDBP	Total 25(OH)D	Bioavailable 25(OH)D	Free 25(OH)D
		HR (95% CI)	HR (95% CI)	HR (95% CI)	HR (95% CI)
**General population**	Zhu 2022 [12]	0.99 (0.79, 1.24)	0.76 (0.60, 0.97)	0.80 (0.63, 1.02)	0.81 (0.64, 1.02)
**Cancer patients**					
Colorectal	Yang 2017 [13]	2.01 (0.92, 4.41)	1.79 (0.90, 3.56)	0.40 (0.08, 1.94)	0.44 (0.24, 0.81)
Colorectal	Yuan 2020 [14]	0.58 (0.37, 0.91)	0.57 (0.34, 0.96)	1.26 (0.77, 2.06)	0.90 (0.68, 1.20)
Liver	Fang 2020 [15]	-	0.97 (0.72, 1.31)	0.69 (0.51, 0.93)	1.35 (0.83, 2.19)
Lung	Turner 2013 [16]	0.10 (0.01, 1.06)	-	-	-
Lung	Anic 2014 [17]	0.95 (0.71, 1.27)	1.18 (0.89, 1.56)	-	-
	**Subtotal**	**0.84 (0.42, 1.67)**	**1.02 (0.70, 1.48)**	**0.83 (0.49, 1.41)**	**0.84 (0.46, 1.51)**
**Other patients**					
**All studies**		**0.90 (0.63, 1.30)**	**0.94 (0.70, 1.26)**	**0.81 (0.63, 1.05)**	**0.84 (0.59, 1.19)**

Abbreviations: CI: confidence interval; HR: hazard ratio; VDBP: vitamin D-binding protein.

## Supplementary Materials

The following supporting information can be downloaded at: https://www.mdpi.com/article/10.3390/nu14193894/s1, Figure S1: Funnel plots for evaluating potential publication bias for associations of VDBP, total, bioavailable, and free 25(OH)D with all-cause mortality; Figure S2: Funnel plots for evaluating potential publication bias for associations of VDBP, total, bioavailable, and free 25(OH)D with cancer mortality; Table S1: Search strategy in PubMed and Web of Science; Table S2: Risk of bias assessment for eligible studies by using Newcastle-Ottawa Scale.
