# Supplementary material for: Vitamin D-Binding Protein, Bioavailable, and Free 25(OH)D, and Mortality: A Systematic Review and Meta-Analysis
Source: Nutrients. 2022 Sep 20;14(19):3894. doi: 10.3390/nu14193894 (PMC9571577; doi:10.3390/nu14193894)
Supplement: Supplementary file 1 [file nutrients-14-03894-s001.zip › nutrients-1899235-supplementary.pdf]

Supplementary materials

Supplemental Table S1. Search strategy in PubMed and Web of Science.

Supplemental Table S2. Risk of bias assessment for eligible studies by using Newcastle-Ottawa Scale.

Supplemental Figure S1. Funnel plots for evaluating potential publication bias for associations of VDBP, total, bioavailable, and free 25(OH)D with all-cause mortality.

Supplemental Figure S2. Funnel plots for evaluating potential publication bias for associations of VDBP, total, bioavailable, and free 25(OH)D with cancer mortality.

Supplemental Table S1. Search strategy in PubMed and Web of Science.

1. Vitamin D biomarkers	MeSH: Vitamin D Binding Protein OR Vitamin D-Binding Protein<br>OR All Fields: “vitamin D binding protein” OR “vitamin D-binding protein” OR “VDBP” OR “gc-globulin” OR “bioavailable 25-hydroxyvitamin D” OR “bio-available 25-hydroxyvitamin D” OR “bio-available 25(OH)D” OR “bioavailable 25(OH)D” OR “bioavailable 25(OH) vitamin D” OR “bio-available 25 (OH) vitamin D” OR “bioavailable vitamin D” OR “bio-available vitamin D” OR “bioavailability of vitamin D” OR “vitamin D bioavailability” OR “free 25-hydroxyvitamin D” OR “free 25(OH)D” OR “free 25(OH) vitamin D” OR “free vitamin D” OR “albumin-bound 25-hydroxyvitamin D” OR “albumin-bound 25(OH)D” OR “albumin-bound vitamin D”
2.Mortality	MeSH: Mortality OR Survival OR Death<br>OR All Fields: “mortality” OR “survival” OR “death” OR “deaths”
3.Dates	Inception-2022/05/27

Footnotes: MeSH terms were only used for systematic search in PubMed.

Supplemental Table S2. Risk of bias assessment for eligible studies by using Newcastle-Ottawa Scale.

Study	Selection				Comparability	Outcome			Total score
	Representativeness of the exposed cohort	Selection of the non-exposed cohort	Ascertainment of exposure	Demonstration that outcome of interest was not present at start of study	Comparability of cohorts on the basis of the design or analysis	Assessment of outcome	Was follow-up long enough for outcomes to occur	Adequacy of follow up of cohorts	
**General population**									
Dejaeger 2021 [11]	1	1	1	1	2	1	1	1	9
Zhu 2022 [12]	1	1	1	1	2	1	1	1	9
**Cancer patients**									
Yang 2017 [13]	1	1	1	1	1	1	1	0	7
Yuan 2020 [14]	1	1	1	1	2	1	1	1	9
Fang 2020 [15]	1	1	1	1	2	1	1	0	8
Turner 2013 [16]	1	1	1	1	1	1	1	0	7
Anic 2014 [17]	1	1	1	1	2	1	1	0	8
Peng 2022 [18]	1	1	1	1	2	1	0	1	8
Chen 2020 [19]	1	1	1	1	1	1	0	0	6
**Other patients**									
Yu 2018 [20]	1	1	1	1	2	1	1	0	8
Persson 2015 [21]	1	1	1	1	2	1	1	1	9
Subramanian 2022 [22]	1	1	1	1	1	1	0	0	6

Footnotes: The studies were listed based on ICD-10 codes of diseases among the participants.

Supplemental Figure S1. Funnel plots for evaluating potential publication bias for associations of VDBP, total, bioavailable, and free 25(OH)D with all-cause mortality.

VDBP

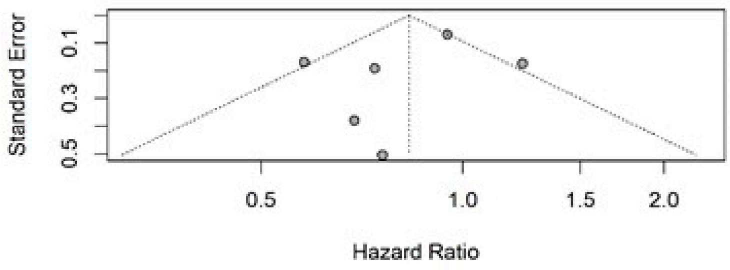

Total 25(OH)D

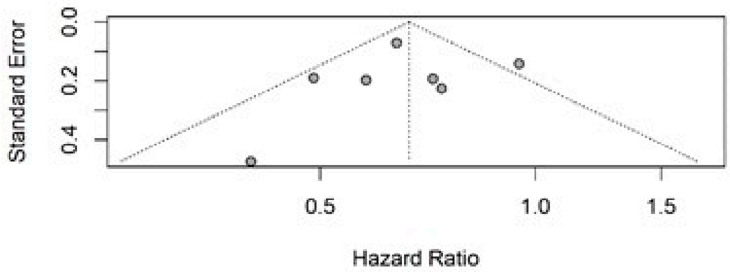

Bioavailable 25(OH)D

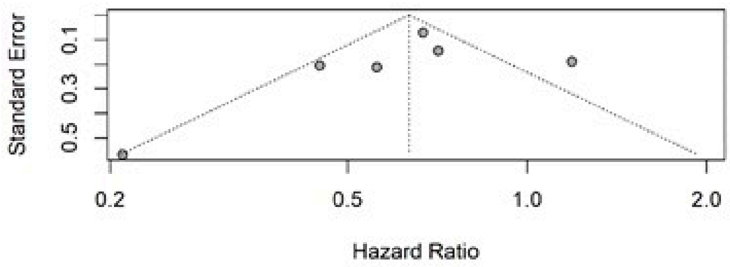

Free 25(OH)D

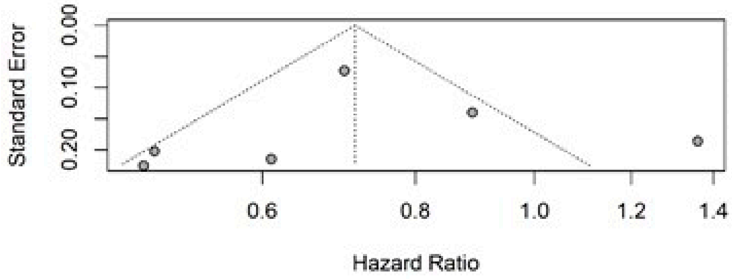

Abbreviations: VDBP: vitamin D-binding protein.

Supplemental Figure S2. Funnel plots for evaluating potential publication bias for associations of VDBP, total, bioavailable, and free 25(OH)D with cancer mortality.

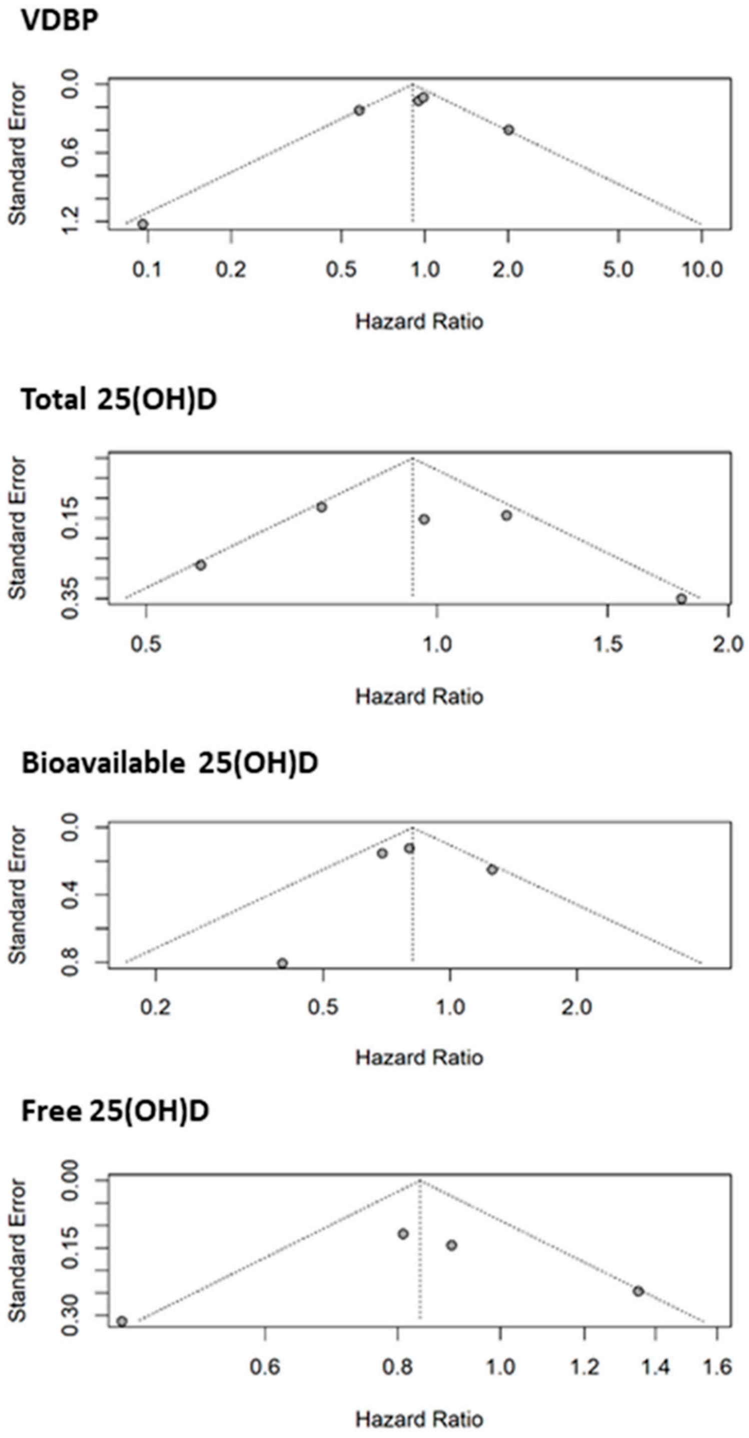

Abbreviations: VDBP: vitamin D-binding protein.
